# Factors influencing regular physical exercise among the elderly in residential care facilities in a South African health district

**DOI:** 10.4102/phcfm.v10i1.1493

**Published:** 2018-04-11

**Authors:** Abiodun A. Aro, Sam Agbo, Olufemi B. Omole

**Affiliations:** 1 Department of Family Medicine, University of the Witwatersrand, South Africa

## Abstract

**Background:**

Physical exercise plays an important role in healthy ageing, but the elderly do not engage in it regularly.

**Methods:**

In this cross-sectional study, we sampled 139 residents of residential care facility. A questionnaire was used to obtain information on participants’ demography, health problems, nature, motivators and barriers to exercise. Chi-square test examined the relationship between participants’ characteristics and their engagement in regular exercise.

**Results:**

Of the 139 participants, the majority were females (71.9%), white people (82.7%), aged 70 years or more (70.5%), had at least one health problem (85.6%) and were overweight or obese (60.4%). Approximately 89.2% engaged in some form of physical activities but only 50.3% reported engaging regularly. Participant’s knowledge of the benefits of regular physical activities, opportunities to socialise, encouragement by health care workers and availability of exercise facilities and trainers promote regular physical exercise. Barriers to regular exercise included poor health status, lack of knowledge of the benefits of regular physical activities, lack of opportunities to socialise, lack of encouragement by health care workers and unavailability of exercise facilities and trainers. Factors that predicted exercise were age 60–69 years (*p* = 0.02), being Afrikaans speaking (*p* = 0.04) and completing high school (*p* = 0.03).

**Conclusion:**

A significant proportion of the elderly do not engage in regular physical exercise, and this behaviour is influenced by personal health status and systems-related motivators and barriers.

## Introduction

Regular exercise delays the onset of chronic diseases and disabilities, and facilitates their control.^1^ Not only is there a reduction in cardiovascular risks through the lowering of blood pressure, low-density lipoprotein (LDL) and triglycerides, there are also increases in high-density lipoprotein (HDL), insulin sensitivity, fibrinolysis and arterial wall compliance.^2^ In the musculoskeletal system, regular exercise in the elderly has been shown to enhance gait and balance, leading to a reduction in the incidence of falls.^2^ Even regular exercise or physical activities initiated late in life have been reported to reduce morbidity and mortality associated with sedentary lifestyle.^3^ Recognising these benefits, the World Health Organization (WHO) recommends moderate intensity activity lasting at least 30 min a day, for at least 5 days a week or 150 min a week for the elderly to stay physically active.^4^ However, despite the benefits of regular physical activity, reports from elsewhere suggest that the elderly do not exercise regularly and that engagement in physical activities progressively declines with age.^5^

There is overwhelming evidence in the literature that poor engagement in physical activities increases the risk of ill health, chronic diseases, gait and balance problems, falls and loss of functional independence in the elderly.^1,4,5,6^ Physical inactivity has also been identified by the WHO as the fourth leading risk factor for global mortality, accounting for 6% of all deaths globally in its report of 2010.^4^ In responding to the prevalent poor engagement of the elderly in physical activities, the South African National Department of Health in alignment with the WHO recommendations recommends that the elderly with stable medical condition should engage in low intensity exercise for at least 30 min a day, at least 3 days a week.^7^ Those who are too frail to stand are encouraged to engage in arm rowing and other rhythmic upper body movements for the same recommended period of time.

Several factors have been reported in the literature to explain the declining engagement of the elderly in regular physical activities.^3,7^ These include cultural background, marital status, level of educational attainment, health status and misconceptions that the elderly cannot and should not exercise. Although these factors have mostly been reported from studies elsewhere, the differences in contexts make direct extrapolation to South Africa a challenge. One of the few available studies in South Africa^8^ reported that although residential care facility (RCF) residents have positive attitudes towards physical exercise, there were substantial gaps in their knowledge of the effects of exercise on health and in information provided by health care providers on exercise. Local data on motivators and barriers to regular exercise are lacking, and yet, such information is essential for developing adequate response to the scourge of physical inactivity among the elderly in South Africa. This is more important noting the increasing ageing population in South Africa and the accompanying burden of chronic diseases.^9^

Residential care facilities provide a well-defined physical environment to gain access to the elderly population and a starting point from which an understanding of the factors influencing regular physical exercise can be gained. The aim of this study was therefore to explore socio-demographic and clinical factors that are associated with regular exercise among residents of RCFs in Ekurhuleni health district, Gauteng Province.

## Methods

### Study design and research setting

This was a cross-sectional study conducted during 2012 in seven RCFs in the southern sub-district of Ekurhuleni health district, Gauteng Province, South Africa. According to the 2011 Census report,^10^ this district had an estimated population of 3.2 million people spread over three sub-district areas. The southern sub-district area where the study was conducted had a population of approximately 900 000, and it includes townships of Alberton, Germiston, Boksburg, Vosloorus, Katlehong and Thokoza.

### Study population, sample size and sampling methods

The study population consisted 823 elderly people who were residents of the seven RCFs in Ekurhuleni southern sub-district in 2012. Consecutive sampling of all eligible residents was performed in all the RCFs. To participate in the study, residents had to meet the following criteria:

- be 60 years or older in accordance with the concept of an older person in South Africa^11^
- have stayed in the old-age home for at least 6 months, to ensure that participants have stayed long enough at the RCF to be engaged in routine activities
- have intact cognitive function and stable medical condition as dementia may limit residents’ capacities to consent and participate in the study. Dementia was determined as documented in the medical record by the psychiatrist.

Residents with visual and hearing impairments were excluded because of limited capacity to participate, and the resources such as hearing and visual aids could not be provided.

### Instrument and data collection

Data were collected using a structured questionnaire developed *de novo* based on the literature review^1,4,12,13,14,15,16,17,18,19^ and modified based on a pilot study conducted on 10 residents of a RCF in Ekurhuleni eastern sub-district. The English version of the questionnaire was translated into Afrikaans and Zulu by two Zulu- and Afrikaans-speaking health care workers who are well grounded in both the language and culture. These translations were back translated into English to check for agreement with the original questionnaire. The questionnaire collected information on participants’ socio-demography, clinical conditions, motivators, barriers, frequency, types and intensities of physical activities or exercise.

The visits to the RCFs were preceded by a phone call to the facility manager to obtain permission. Each facility was visited at different times for data collection. The questionnaire was administered by the researcher to English-speaking participants, whereas two trained research assistants administered the Zulu and Afrikaans versions to participants who were unable to communicate in English language under the direct supervision of the researcher. Afterwards, the researcher measured the weight and height and calculated the body mass index of the participants. The clinical diagnoses were also extracted from the medical record. Participant’s health status was classified as ‘healthy’ (participants without any health-related problems) or ‘less healthy’ (participants with one or more chronic medical condition). The completed questionnaires were coded immediately to ensure confidentiality and securely kept in a drawer accessible only to the research team.

### Data analysis

Data from the questionnaire were captured unto an MS Excel spreadsheet and later imported into STATA 10 statistical software (version 10.1, copyright 1984–2009 Statacorp, Texas, USA). Analysis was performed with the assistance of a statistician. Descriptive statistics was performed to describe participants’ socio-demography, clinical characteristics, the types, frequencies and intensity of physical activities. Although the ethnicities of the participants were collected in the questionnaire, the native language of the participant was used as proxy for ethnicity.

Physical activities were grouped into three intensity categories as defined by Kistler et al. (low, medium and high).^19^ Exercise was defined as any activity requiring effort that is carried out to sustain or improve health and fitness, and regular exercise is engagement in exercise for at least 150 min per week (at least 30 min in a day and five times per week) according to WHO.^4^ Self-directed exercise such as walking to and from dining room, within the facility, walking up and down the stairs, walking around shopping malls and walking outside was included in the definition of regular exercise and not only formal exercise under the guidance of an instructor. Motivators and barriers to regular exercise were identified from participants’ responses and were grouped into themes. Where necessary, continuous data were reclassified into groups. Bivariate analysis was used to determine statistically significant motivators and barriers to regular exercise. Chi-square log-linear test was used to determine associations between participants’ characteristics and engagement in regular physical activities. Statistical significance was deemed to exist when *p* < 0.05.

### Ethical considerations

Ethics clearance was obtained from the Human Research Ethics Committee of the University of the Witwatersrand (Clearance certificate number M120120). Permission to conduct the study was obtained from the managers of each old-age home and from the Research Ethics Committee of Ekurhuleni health district. Written informed consent was obtained from all participants. Identifying data were coded and not linked to individual participants. All data were treated confidentially and were only accessible to the research team.

## Results

Of the 823 eligible RCF residents, 646 did not meet the inclusion criteria and were thus excluded. Of the eligible 177 residents, 38 declined to participate, leaving 139 participants, all of whom were consecutively sampled in the study (response rate of 78%).

Table 1 shows participants’ socio-demographic characteristics. The mean age was 70.4 years with the majority being females (71.9%) and aged 70 years or older (70.5%). Participants’ health statuses are shown in Table 2. The majority (86%) had one or more health problems such as hypertension, diabetes mellitus type 2 or osteoarthritis. Although the mean body mass index (BMI) was 26.6 kg/m^2^, more than half (60%) of the participants were overweight or obese.

**TABLE 1:** Participants’ characteristics.

Variable	Percentage (*n*)
**Sex**	
Male	28 (39)
Female	72 (100)
**Age (years)**	
60–69	29 (41)
70–79	37 (51)
≥ 80	34 (47)
**Marital status**	
Single	17 (23)
Divorced	13 (18)
Widowed	56 (78)
Married	9 (13)
Separated	5 (7)
**Language background**	
Afrikaans	44 (61)
English	39 (54)
Zulu	5 (7)
Others	12 (17)
**Educational attainment**	
Did not attend school	8 (11)
Completed primary school education	12 (16)
Completed high school education	58 (81)
Had tertiary education	22 (31)

**TABLE 2:** Clinical information of participants.

Variable	Percentage (*n*)
**Presence of health problems**	
No health problem	14 (20)
Has health problems	86 (119)
**Body mass index (BMI) kg/m^2^**	
Normal weight (BMI 19–25)	40 (55)
Overweight (BMI 26–29)	34 (48)
Obese (BMI > 30)	26 (36)

Most participants (92.8%) engage in some form of exercise, but only about 50.3% reported engaging in physical exercise regularly. Table 3 shows that 89.2% of participants engage in low intensity exercises. Table 4 shows factors that were reported to influence regular exercise. Most participants were motivated to engage in regular exercise by the knowledge of the health benefits of physical exercise (98%) followed by the opportunity to socialise during physical exercise (64.2%). In contrast, reported barriers to engaging regularly in physical exercise included poor health status (82.6%) and lacking the knowledge of the health benefits of physical exercise (72.4%).

**TABLE 3:** Types and duration of exercises.

Variable	Percentage
**Types of exercises**	
Low intensity	89
Moderate intensity	14
High intensity	4
No exercise done	7
**Duration of exercise in a week**	
< 150 min/week	40
> 150 min/week	50
Don’t know	2
No exercise done	8

**TABLE 4a:** Motivators to regular exercise.

Motivators	Exercised regularly (*n* = 70)			
	Frequency	Percentage	*X*^2^	*p*
Knowledge of exercise benefits	69	98.5	17.9	< 0.001
Opportunity to socialise	45	64.2	55.5	< 0.001
Encouragement by health workers and relatives	37	52.8	44.4	< 0.001
Availability of trainer and exercise facilities	25	35.7	17.2	< 0.001

**TABLE 4b:** Barriers to regular exercise.

Barriers	Do not exercise regularly (*n* = 69)			
	Frequency	Percentage	*X*^2^	*p*
Poor health	57	82.6	94.1	< 0.001
Lack of knowledge of exercise benefits	50	72.4	67.1	< 0.001
Lack of encouragement	38	55.1	55.0	< 0.001
Lack of interest	27	39.1	33.9	< 0.001
Lack of exercise facility	24	34.7	29.4	< 0.001

As shown in Table 4, in bivariate analysis, all the reported motivators were significantly associated with regular exercise, and all reported barriers were significantly associated with not engaging in regular exercise. However, in the log-linear analysis (shown in Table 5), only age 60–69 years, being of Afrikaans speaking group and completing high school were factors significantly associated with engaging in regular physical exercise.

**TABLE 5:** Socio-demographic and clinical factors in relation to regular exercise.

Variable	Regularly exercised		*p*	*X*^2^
	Frequency	Percentage		
**Gender**			0.90	0.002
Male	19	27.2		
Female	51	72.8		
**Age (years)**			0.02	7.500
60–69	27	38.6		
70–79	19	27.1		
≥ 80	24	34.3		
**Body mass index**			0.08	4.800
18–24.9 kg/m^2^	27	38.6		
25–29.9 kg/m^2^	24	34.3		
≥ 30 kg/m^2^	19	27.1		
**Marital status**			0.20	6.600
Single	10	14.3		
Married	3	4.3		
Separated	2	2.8		
Divorced	9	12.9		
Widowed	46	65.7		
**Educational attainment**			0.03	10.500
Did not attend school	1	1.4		
Attended primary school	9	12.9		
Attended high school	44	62.9		
Has a diploma	12	17.1		
Has a university degree	4	5.7		
**Language background**			0.04	6.600
Afrikaans	33	47.1		
English	30	42.9		
Others	7	10.0		
**Health status**			0.40	0.500
Healthy	10	14.3		
Less healthy	60	85.7		

## Discussion

This study found that about half of the residents in RCFs engage in regular physical activities and only a minority do so regularly. Residents aged 60–69 years, who are Afrikaans speaking, females, with normal body mass index, widowed and attended high school were more likely to engage in regular physical activities. These findings confirm those of previous studies elsewehere^12,13,19,20^ and highlight the enormous missed opportunities that exist in leveraging the health benefits of regular physical exercise for improved health status of the elderly in South Africa.

The finding of this study that younger elderly people were more likely to engage in regular exercise corroborates previous reports^1,21^ and may be explained by reduced engagement in physical exercise because of general increases in morbidity and reduced mobility imposed by degenerative and chronic diseases as age advances.^3^ Considering that exercise slows down the pace of morbidity and promotes healthy ageing,^1,2^ there is a need to intensify efforts at encouraging elderly to participate in regular exercise, especially as age advances.

In this study, participants whose native languages were Afrikaans and English were significantly more likely to exercise regularly probably because they are generally more likely to have higher educational attainments and are therefore more knowledgeable about the benefits of regular exercise – the most reported motivator for engaging in regular exercise in this study. This explanation is further corroborated by the finding of a significant positive association between higher educational attainment and regular exercise, and confirms the proposition of the adapted motivation equation that educating people on the benefits of regular exercise could enhance the perceived importance and therefore the uptake of regular exercise, even where exercise programmes and equipment are not available.^15,22,23,24^ It is therefore important to target elderly RCF residents with low educational attainments with the aim of increasing their knowledge of the health benefits of regular exercise. However, this finding could reflect a class effect, as people with higher education may have access to more physical exercise resources because of economic ability.

Having knowledge of the benefits of exercise alone may not translate into increased uptake of regular exercise. The Cox’s physical activities model suggests that engagement in regular physical exercise is dependent on the interactions of personal, environmental and interpersonal factors.^25^ Factors such as the need for socialisation and the feelings of enjoyment associated with regular exercise play important roles in long-term engagement in physical exercise.^26^ Hence, integrating social components of daily living into exercise programmes may therefore increase exercise uptake, especially that almost two-third of participants reported that having opportunity to socialise motivated them to engage in regular exercise.

The majority of participants in this study were motivated to exercise regularly based on encouragement received from health care workers and relatives, contrary to the assertion that although health care workers (especially doctors) have great influence on their patients, they do not encourage their patients to engage in regular exercise.^20^ Health care workers should see every patient contact as an opportunity to promote physical exercise among the elderly, especially those without spouses or social support (such as widowed),^14,27^ because they are more likely to be lonely and require more encouragement and support from health care workers to engage in regular physical activities.

The provision of exercise facilities and the availability of trainers have been previously associated with increased uptake of regular exercise among the elderly^28^ and support the findings in this study. However, although subsidised by the government, only three of the seven old-age homes studied had exercise programmes that consisted mainly of stretching exercises and riding on a stationary bicycle. This may have negatively affected the residents’ ability to exercise. Although the provision of exercise facilities and trainers may incur additional financial expenses, the opportunity to create fora for socialising, the potential to increase the uptake of physical exercise and the resulting improved health status of residents of RCFs^13,28,29,30^ should all justify investing in procuring these facilities. Although RCFs do not need a physical trainer to have an exercise programme, a trainer could provide health education on the health benefits of regular exercise and encourage RCFs residents to engage in physical activities that are commensurate with their level of physical function. Furthermore, issues such as ensuring a safe neighbourhood, adequate physical space, dedicated exercise rooms and exercise equipment may all encourage residents of RCFs to engage in regular exercise and need to be looked into when developing physical exercise programmes in RCFs.

Previous studies have shown that poor health status is a common barrier to regular exercise among the elderly.^1,30^ However, most participants with health problems or poor health in this study still engaged in one form of physical activity or the other, albeit not regularly, suggesting that poor health in itself may not be an absolute barrier to exercise, and in the context of a good knowledge of the benefits of regular exercise, poor health status could paradoxically be a motivation for engaging in regular exercise. Belza and colleagues^23^ noted that participants in their study considered poor health as a motivation for regular exercise and even considered physical activities more important than taking medication. However, they alluded to the influence of the knowledge of health benefits of exercise on engagement in physical activities despite poor health, especially that this relationship may be mediated by the level of education.^23,30^ In a similar vein, we reason based on the Geelen and Soons adapted motivation equation^24^ that if engaging in regular exercise as a health maintenance and promotion strategy is perceived more important than the cost of doing so (e.g. pain or fear of falling), the elderly are more likely to engage in regular exercise despite poor health status.^13,28^ Therefore, optimising the management of chronic diseases and pain among the elderly (that often limit physical mobility) can reduce perceived costs and increase their participation in regular physical exercise.^30^

The lack of interest in physical activities reported as a barrier to regular physical exercise is most likely multi-factorial and has been suggested to be because of personal, interpersonal and environmental influences by Cox’s model.^25^ Personal factors such as past life experiences, poor understanding of the benefits of exercise and poor self-efficacy may influence the lack of interest reported by participants.^23,31^ Perceived high costs of engaging in exercise, lack of encouragement by health workers and relatives, and unfavourable environmental conditions could all create lack of interest in engaging in regular exercise in the elderly.^17,18,27^

Physical inactivity is a significant and leading risk factor for morbidity and mortality, and in the absence of robust data to inform interventions among the elderly in South Africa, this study provides some insight into what motivates or hinders their engagement in regular physical exercise. This study therefore has the potential to inform further studies in South Africa and in similar contexts. However, this study has potential for certain limitations and potential biases.

Firstly, the sample is not random nor nationally representative, and care should be taken not to generalise the study findings. However, this sample is a good reflection of the population in the urban South African RCFs in that non-Caucasians are culturally less acceptable of being placed in RCFs^21^ and considering socio-economic disparities may even be less likely to afford the costs of living in RCFs.

Secondly, this study was based on self-reports and is therefore prone to information and social desirability biases.

Thirdly, the eventual small sample might have under-powered the study to the end that some otherwise statistically significant differences might have been missed.

In conclusion, despite the benefits, this study reiterates reports that the elderly in RCFs do not engage in regular physical activities. Furthermore, it has identified some personal, health status and systems-related factors that influence the engagement of the elderly in regular physical exercise. Interventions aimed at increasing the uptake of regular physical exercise among RCFs’ residents should promote health education to improve the elderly’s knowledge of the benefits of regular exercise, ensure that health care providers encourage the elderly to exercise regularly, create opportunities for socialisation, integrate physical exercise into daily activities, ensure optimal control of chronic and degenerative diseases, and enact policies that mandate the provision of exercise facilities and equipment in RCFs. In view of the limitations of this study, larger and nationally representative quantitative designs and in-depth qualitative studies are needed to gain better understanding of physical inactivity among the elderly in South Africa.
